# Reevaluating Gold(I) Thiophilicity in Reduced Dielectric Environments: IRMPD Spectroscopy and Theoretical Investigation of Auranofin–Glutathione Complexes

**DOI:** 10.1155/bca/5944632

**Published:** 2026-10-19

**Authors:** Roberto Paciotti, Susanna Grasso, Cecilia Coletti, Nazzareno Re, Giel Berden, Jos Oomens, Charles Desfrançois, Simonetta Fornarini, Davide Corinti, Maria Elisa Crestoni

**Affiliations:** ^1^ Dipartimento di Farmacia Università G. D’Annunzio Chieti-Pescara Via dei Vestini 31 Chieti I-66100 Italy; ^2^ Dipartimento di Chimica e Tecnologie del Farmaco Sapienza Università di Roma, I-00185 Roma Italy; ^3^ HFML-FELIX Toernooiveld 7 6525ED Nijmegen the Netherlands; ^4^ Institute for Molecules and Materials Radboud University Heyendaalseweg 135 6525AJ Nijmegen the Netherlands ru.nl; ^5^ Laboratoire de Physique des Lasers CNRS Université Sorbonne Paris Nord 93430 Villetaneuse France cnrs.fr

**Keywords:** auranofin, DFT, EAD, gold(I) complexes, GSH, IR ion spectroscopy

## Abstract

The coordination of gold(I) to glutathione (GSH) is a fundamental process in the medicinal chemistry of auranofin and related therapeutics. Utilizing infrared multiple‐photon dissociation (IRMPD) spectroscopy, electron‐activated dissociation (EAD), and quantum‐chemical calculations, we characterize the gas‐phase structure of the complex [(Et_3_P)AuGSH]^+^. Our results reveal that in the gas phase, the NH_2_ group of the N‐terminal glutamate residue of GSH serves as a competitive coordination site, challenging coordination by the canonical cysteine thiol, with both isomers coexisting in comparable abundances. This finding suggests that in microenvironments characterized by a reduced dielectric constant, the GSH–Au(I) complex might also exist as an N‐bound isomer with a free thiol, which may significantly affect its biological reactivity and functions. Moreover, considering that the dielectric constant within buried protein active sites is substantially lower than that of bulk water, this N‐binding motif identifies the unprotonated N‐terminus as a plausible target for metalation, even in the presence of free thiols. These insights offer a broader perspective on the competitive binding landscape of gold(I) drugs. As a future direction, we hypothesize potential implications for the inhibition of enzymes with N‐terminal catalytic groups, which are crucial for tumor progression, such as nucleophile (Ntn) hydrolases.

## 1. Introduction

Auranofin (AF) (1‐Thio‐β‐D‐glucopyranosyltriethylphosphine gold‐2,3,4,6‐tetraacetate, Figure 1A) is a clinically approved gold(I)–phosphine complex originally indicated for rheumatoid arthritis. Recent studies have explored the drug repurposing potential of AF, highlighting its efficacy as an anti‐inflammatory, antimicrobial, and antineoplastic agent [1, 2].

**FIGURE 1:**
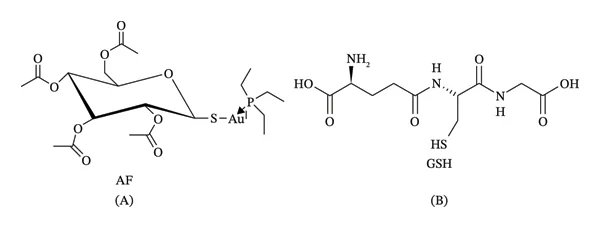
Structure of (A) auranofin (AF) and (B) glutathione (GSH).

AF exerts its pharmaceutical action through a mechanism that is not yet fully elucidated, based on the inhibition of thioredoxin reductase (TrxR) and thioredoxin glutathione reductase (TGR) enzymes, which are involved in cellular redox reactions and quenching of intracellular reactive oxygen species (ROS). The Au(I) contained in AF has a high affinity for thiol and selenol groups, forming very stable adducts with molecules containing those sites [3, 4]. In this regard, the binding and inhibition of TrxR, which contains selenocysteine (Sec) in the active site, disrupts the cell’s ability to mitigate oxidative stress, ultimately triggering apoptosis.

Crucial to this redox landscape is glutathione (GSH) (Figure 1B), a tripeptide formed by γ‐glutamate–cysteine–glycine (γ‐Glu–Cys–Gly), representing the most abundant low‐molecular‐weight thiol in the intracellular environment [5]. GSH plays a critical role in the neutralization of ROS and free radicals, as well as being involved in metal detoxification and iron metabolism. Given the high affinity of gold(I) for sulfur ligands, GSH–gold interactions are well‐documented [6]. In fact, the stable Au–S bond was used to develop GSH‐covered Au nanoparticles (AuNPs) for several purposes, such as to produce antiamyloidogenic agents [7] or to stabilize AuNPs conjugated with chemotherapeutic agents (doxorubicin, cytarabine, and gemcitabine) for the treatment of several tumors [8]. Notably, the cytotoxic efficacy of AF is significantly increased by GSH depletion [9–11] or dysregulation [12], suggesting a protective or competitive role for GSH. Such depletion likely intensifies AF‐induced cytotoxicity by diminishing the efficiency of the cellular antioxidant system. Moreover, GSH may directly sequester Au(I) affecting the drug reactivity against TrxR or other redox‐active enzymes. One such target is GSH peroxidase (GPX), which contains a Sec residue in its active site and utilizes GSH as a substrate [13]. In this instance, AF exerts its inhibitory effect through the formation of a (glutathionate–gold(I)–SecGPX) ternary complex [14, 15].

Despite its significance, the exact structural nature of the Au(I)–GSH interaction at a molecular level remains elusive. A powerful spectroscopic technique apt to study the structural features of metal‐containing ions is infrared multiple‐photon dissociation (IRMPD) spectroscopy, which is based on irradiating mass‐selected ions trapped in a mass spectrometer by a tunable infrared laser, thus inducing photofragmentation when the photon energy is resonant with one of the ion’s vibrational modes [16]. The infrared spectrum of the selected ion can be obtained by plotting the photofragmentation yield as a function of the photon energy. Finally, quantum‐chemical calculations, generally employing density functional theory (DFT), are used for vibrational and structural assignment. This dual approach was widely applied to metal complexes of biological relevance [17], including those containing Au(I) [18–21], GSH [22, 23] and its complexes with several metal ions [24]. Moreover, we recently applied this IRMPD/DFT framework to elucidate the binding of AF fragments to isolated Cys and Sec residues, demonstrating the value of this approach in elucidating not only the structure but also the reactivity of biological complexes containing Au(I) [25].

We found that the sampled population of the [(Et_3_P)AuCys]^+^ ion, the adduct formed by Cys and AF, comprises both N‐ and S‐bound isomers. Crucially, their relative ratio depends on the dielectric constant of the starting solution, with low‐dielectric environments stabilizing the N‐bound isomer (where the α‐NH_2_ coordinates the Au(I) center) despite gold(I)’s well‐known thiophilicity. This was a significant finding, as the N–Au binding motif had previously only been observed in whole proteins lacking free Cys and His residues [27], such as thaumatin (Figure 2) [26].

**FIGURE 2:**
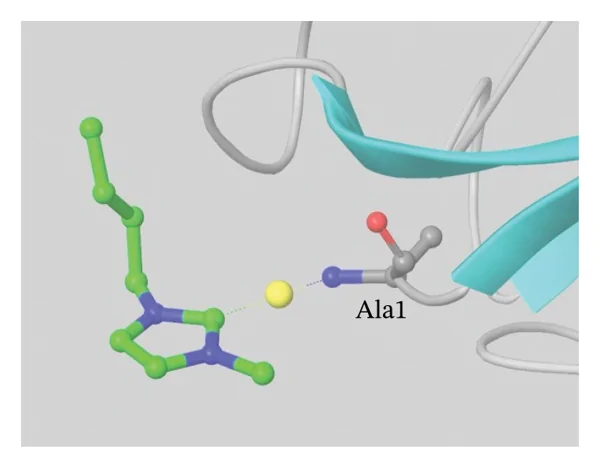
Structural representation of the N–Au binding motif in thaumatin, a protein devoid of free Cys and His residues. The Au(I)–carbene compound coordinates the N‐terminal α‐NH_2_ group of Alanine 1 (Ala1) to form the adduct (adapted from PDB ID: 5JVX [26]). The carbon atoms of the Ala1 residue and the carbene ligand are shown in gray and green, respectively, whereas the Au, N, and O atoms are represented in yellow, blue, and red.

In the present study, we aim to extend our investigation to the reaction product of AF with GSH, [(Et_3_P)AuGSH]^+^. By applying our combined IRMPD/DFT approach, this work has two main objectives:

i. To clarify the binding motif between Au(I) and GSH, assessing the influence of the solvent environment on complex stability.
ii. To evaluate the viability of Au(I) coordination to the α‐NH_2_ moiety even in the presence of a competing free –SH group. Herein, GSH serves as a model system of a protein: as a small peptide with native N‐ and C‐termini, it is structurally more representative than an isolated Cys, yet small enough to permit highly accurate DFT calculations.

Thus, by characterizing the structural motifs of the [(Et_3_P)AuGSH]^+^ complex, we aim to provide novel molecular‐level insights into how the solvent can modulate the metalation of GSH nucleophilic centers, with profound implications for Au(I)–protein interactions and ensuing pharmacological applications.

## 2. Experimental Section

### 2.1. Materials

AF and GSH were purchased from Merck s.r.l., Milan, Italy, and used without further purification. Aqueous solutions of AF and GSH at a concentration of ca. 10^−3^ M were mixed in a 1:1 ratio and finally diluted in water/methanol 1:1 to reach a final concentration of 10^−5^ M of each analyte.

### 2.2. HRMS and Electron‐Activated Dissociation (EAD) Experiments

High‐resolution mass spectrometry (HRMS) experiments were conducted on a ZenoTOF 7600 mass spectrometer (SCIEX), equipped with a Zeno trap and an EAD cell. The [(Et_3_P)AuGSH]^+^ precursor ion was generated via electrospray ionization (ESI) and mass‐selected in the Q1 quadrupole. The ions were then introduced into the EAD cell, where they were either only submitted to collision‐induced dissociation (CID) or made to interact with a kinetic energy (KE)–controlled electron beam to perform EAD. The EAD parameters were optimized to maximize the yield of informative fragment ions: the electrospray current was set to 3500 nA, the electron KE was maintained at 10 eV, and the reaction time was 30 ms. The resulting product ions were analyzed in the high‐resolution time‐of‐flight (TOF) analyzer. External mass calibration was performed prior to each run via the integrated calibrant delivery system connected to the ESI calibration probe of the ZenoTOF OptiFlow source. Automated calibration managed by the SCIEX OS software ensured a maximum mass error of 2 ppm for the calibrant solution (X500 ESI Positive Calibration Solution) and a resolving power of ca. 30,000 at 500 m/z. Given the low intensity of certain minor fragment ions, a tolerance window of 10 ppm was applied for molecular formula assignment using the “Formula Finder” tool within SCIEX OS. All assigned fragment ions were identified considering the elemental composition of the precursor ion and based on the lowest mass error, which remained under 6 ppm across all cases. The use of the Zeno trap provided a significant duty‐cycle enhancement, enabling the detection of low‐abundance diagnostic fragments [28]. Reproducibility of the EAD and CID experiments was verified across independent runs, showing negligible variation in spectral features.

### 2.3. IRMPD Spectroscopy Experiments

IR spectroscopy experiments on mass‐selected ions were carried out at the Free Electron Laser for Infrared eXperiments (FELIX) facility (Nijmegen, The Netherlands) [29] using a commercial 3D quadrupole ion trap mass spectrometer (Bruker amaZon speed ETD) that had been modified to enable interaction between trapped ions and the FEL radiation [30]. The complexes of interest were mass‐selected and irradiated with a single IR macropulse from the FEL, inducing frequency‐dependent IRMPD. Each macropulse (∼7 μs in duration) consists of a train of ∼5 ps‐long micropulses separated by 1 ns.

For the purpose of this study, FELIX was operated in the structurally diagnostic fingerprint region (700–1900 cm^−1^), targeting the most intense and informative vibrational features, delivering pulse energies in the range of 30–90 mJ per macropulse with a bandwidth of approximately 0.5% of the IR frequency. Spectra were acquired with a step size of 5 cm^−1^, averaging six replicate mass spectra at each irradiation frequency. Measurements were performed at different levels of laser pulse energy attenuation to prevent excessive depletion of the precursor ions and to reduce the formation of fragment ions with m/z values below the low‐mass cutoff of the mass spectrometer [31]. The final IRMPD spectrum was obtained by combining data collected at different attenuation settings, ensuring an optimal spectral profile that accurately represents the system while avoiding the loss of spectroscopic information.

The IRMPD spectra were visualized by plotting the photofragmentation yield, *R* (*R* = −ln[*I*_*P*_/(*I*_*P*_ + Σ*I*_*F*_)]), as a function of the photon wavenumber, where *I*_*P*_ and *I*_*F*_ denote the abundances of the precursor ion and fragment ions, respectively [32]. A linear correction accounting for the frequency‐dependent variation in laser pulse energy was subsequently applied to the yield values [33].

### 2.4. Computational Methods

A computational study was performed to identify the most stable isomers and conformers of [(Et_3_P)AuGSH]^+^ and to support the interpretation of the experimental IRMPD spectrum. As shown in Figure 3, GSH can coordinate Au(I) through various functional groups of the Cys (c), Glu (e), and Gly (g) residues, including SH, N‐terminal α‐NH_2_, C=O, and the carboxyl function (COOH). These coordination motives lead to the corresponding **cSH**, **eN**, **cCO**, **eCOOH**, and **gCOOH** isomers (where the lowercase prefixes “c,” “e,” and “g” denote Cys, Glu, and Gly residues, respectively). Coordination to the SH or COOH groups significantly increases their acidity, facilitating proton transfer to the Glu amine group and generating zwitterionic isomers, designated as **cS**, **eCOO**, and **gCOO** (Figure 3).

**FIGURE 3:**
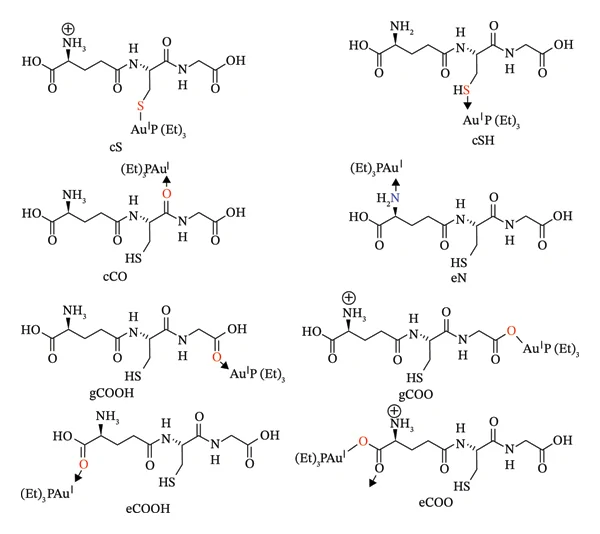
2D structures of [(Et_3_P)AuGSH]^+^ isomers investigated in this study, along with their corresponding labels (see the text for details).

To identify the lowest‐energy conformers of the [(Et_3_P)AuGSH]^+^ complex, a conformational search was performed for each isomer at the DFT tight‐binding level of theory (GFN2‐xTB method) [34, 35], using the iMTD‐GC algorithm implemented in the CREST software [36, 37]. A clustering analysis was then carried out using the CREGEN procedure available in CREST.

Representative low‐energy structures were preliminarily optimized at the B3LYP/6‐311+G(d,p) level of theory, using the LANL2DZ pseudopotential for Au and empirical D3 dispersion corrections with Becke–Johnson damping functions, D3(BJ), to improve the description of dispersion interactions [38]. The geometries of the lowest‐energy conformers for each isomer were refined at the same level of theory, adopting a basis set referred to as BS1, consisting of 6‐311+G(2df,pd) for H, C, N, and O atoms, 6‐311+G(3df) for P and S, and the LANL2TZ‐f pseudopotential for Au [39]. Thermochemical parameters, including zero‐point energy (ZPE), thermal corrections, and entropies, were calculated at this level together with the harmonic vibrational frequencies used for the interpretation of experimental IRMPD spectra. Theoretical harmonic frequencies were scaled by a factor of 0.98 in the 700–1900 cm^−1^ range, and all calculated spectra were convoluted assuming a Gaussian profile with a full width at half maximum (FWHM) of 15 cm^−1^, in agreement with previously reported values [25].

The relative free energy of **eN-1** and **cS-1** in methanol and water was also assessed by performing geometry optimization and frequency calculations at the B3LYP‐D3(BJ)/BS1 level of theory using the Onsager method [40]. Dielectric constants of 78.36 and 32.61 were adopted for water and methanol, respectively. Although this approach is, in principle, less accurate than continuum solvation models such as CPCM [41, 42] or SMD [43], the latter are known to produce artifacts in the definition of the solute cavity for large molecules, thus failing to reproduce accurately the space accessible to the solvent [44]. Indeed, in a recent study on similar systems [25], we found that the Onsager method provided results in closer agreement with experiment than CPCM.

All quantum chemical calculations were performed using the Gaussian 09 package [45].

## 3. Results and Discussion

### 3.1. Identification and MS Characterization of [(Et_3_P)AuGSH]^**+**^

The sample solution containing AF and GSH was mass analyzed in positive ion mode using the ZenoTOF 7600. The recorded mass spectrum (Figure S1 in the SI) shows the presence of a signal at m/z 622.1399 corresponding to an ion where the thioglucose moiety of AF is substituted by GSH, thus yielding [(Et_3_P)AuGSH]^+^. Comparison of the obtained accurate mass with the calculated exact mass shows an error of 1.7 ppm, thus confirming the assigned molecular formula C_10_H_17_N_3_O_6_SAuC_6_H_15_P. Species similarly formed by substitution in the presence of Cys, methionine, and methyl cysteine were previously identified by MS [25, 46]. In agreement with previous results, Figure S1 presents signals pertaining to AF, i.e., [AF + H]^+^ at m/z 679, and the corresponding sodiated and potassiated complexes at m/z 701 and 717, respectively, and [(Et_3_P)_2_Au]^+^ at m/z 433.

GSH is a tripeptide (γ‐Glu–Cys–Gly) with several functional groups potentially capable of binding Au(I), such as the SH, C=O, NH_2_, and COOH groups, which belong to different residues, thus leading to several possible isomeric complexes with gold (Figure 3). A previous study on the complexes resulting from the reaction of AF with Cys and Sec demonstrated that, when interacting with cysteine, Au(I) can bind both the S‐atom of the thiol group and the N‐atom of the α‐NH_2_ group with comparable efficacy [25]. In this study, gas‐phase fragmentation methods including CID and EAD are thus applied to gather information about the binding motifs. While CID is commonly used for structural elucidation of ions, the potential for metal‐center migration during slow collisional activation often complicates structural assignments. On the other hand, EAD typically proceeds via a nonergodic mechanism consisting of a rapid, radical‐driven cleavage of the peptide backbone, which occurs on a faster timescale, avoiding significant intramolecular rearrangements [47]. Therefore, EAD‐specific channels where the [(Et_3_P)_2_Au]^+^ moiety remains bound to a specific amino acid residue can contribute to clarifying the precursor ion’s original coordination state.

The CID and EAD tandem mass spectra of [(Et_3_P)AuGSH]^+^ (m/z 622.1407) (see Figures S2 and S3, respectively) exhibit complex dissociation with distinctive fragments. Enlargements of the fragmentation mass spectra are reported in Figures S4–S6, showing selected regions of the spectra comprising EAD‐specific fragments of interest. The list of fragments and their attributed molecular formula is reported in Table S1, while Schemes S1 and S2 show tentative assignments of their structure with reference to the plausible starting structure. Figures S7–S11 show details on the molecular formula assignments by elemental composition analysis on selected fragments. Most of the fragments are observed in both CID and EAD mode, while the few which are EAD‐specific are appropriately called out in the text. An example of the latter set is the fragment at m/z 621.1297 obtained by hydrogen atom loss, which is consistent with the radical nature of EAD.

Specific fragments which provide suggestions for the binding motifs (Scheme 1) include the EAD‐specific radical ion at m/z 475.0640 and the fragments at m/z 493.0984 and 476.0718 (Figure S6), which are related to the loss of the glutamyl residue and may suggest that the gold–phosphine unit is coordinated to the cysteine thiolate. In fact, when interacting with the amino acids Cys and Sec, Au(I) did not show any tendency to bind to the carboxylic group, the other possible competing nucleophile in the remaining ion (see Scheme 1 and S1). Further confirmation is the presence in the EAD spectrum of an ion at m/z 401.0398 (Figure S4), attributable to [(Et_3_P)Au(SC_2_H_3_CO)]^+^. Concurrently, an alternative fragmentation pathway emerges that may indicate nonsulfur coordination. The key EAD‐specific fragment at m/z 461.1263 corresponds to a *c*_1_ ion formed through a nonergodic cleavage mechanism. This direct, fast backbone cleavage suggests localization of the gold center to the N‐terminal glutamic acid residue, alongside the product ion at m/z 417.1365 (Schemes 1 and S2). The presence of a species in which the S‐atom is not coordinated to Au(I) is further supported by the neutral loss of H_2_S (m/z 588.1532). While H_2_S loss is a vibrationally activated channel wherein rearrangements can occur, it nevertheless provides complementary evidence for an active ion population featuring an exposed, free thiol group.

**SCHEME 1:**
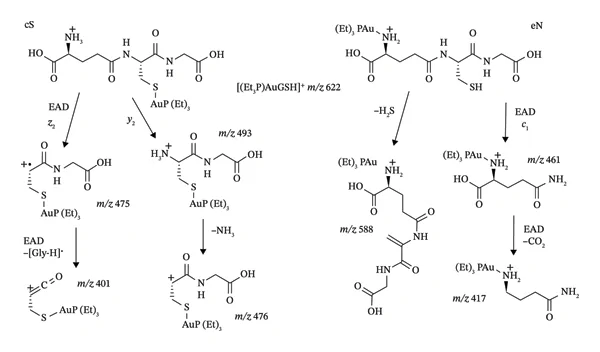
Simplified dissociation pathways of the [(Et_3_P)AuGSH]^+^ complex (m/z 622). The pathway on the left illustrates the **cS** isomer, with the diagnostic *z*_2_ and *y*_2_ fragments. The pathway on the right illustrates the **eN** isomer, characterized by the *c*_1_ fragment and H_2_S loss.

The observation of these mutually exclusive fragmentation markers, specifically the retention of gold on the Cys–Gly backbone (presumably a **cS** isomer) versus the localization of gold on the Glu residue, provides evidence for isomeric heterogeneity in the [(Et_3_P)AuGSH]^+^ion population. The identity of all fragment ions was confirmed with a mass accuracy within 5 ppm, with the sole exception of the radical ion at 621.1332 m/z showing an error in ppm of 5.6 (Table S1).

### 3.2. Structural and Vibrational Features of [(Et_3_P)AuGSH]^**+**^

Gas‐phase dissociation experiments have suggested a complex chemistry for the AF reaction with GSH involving not only the high‐Au‐affinity thiol group but also the N‐atom of the glutamic acid amino group. To further elucidate the structural features of the [(Et_3_P)AuGSH]^+^ complex, a theoretical investigation was performed to identify the lowest‐energy structures and support the interpretation of experimental signatures.

DFT calculations indicate that the most stable structures are **cS-1** and **eN-1**. These species belong to different isomer families but are characterized by nearly identical free energy values, as reported in Figure 4 and Table S2.

**FIGURE 4:**
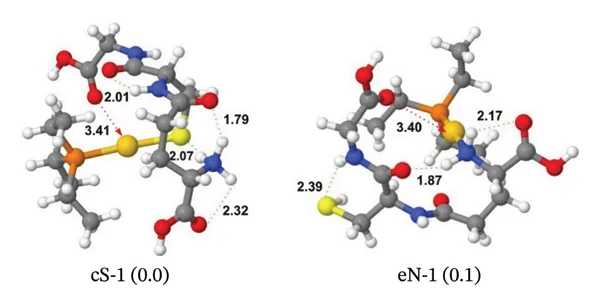
Optimized geometries of the most stable isomers and conformers of the [(Et_3_P)AuGSH]^+^ ion, computed in the gas phase at B3LYP‐D3(BJ)/BS1 level of theory. Free energy values relative to **cS-1** are reported in parentheses in kJ mol^−1^. Hydrogen bonds and gC=O…Au(I) distances (Å) are indicated by green and red dotted lines, respectively.

In **cS-1**, the thiolate function of Cys binds directly to the metal center, with the proton transferred to the Glu α‐amino group (forming NH_3_^+^), resulting in a zwitterionic form of GSH. Specifically, this structure is stabilized by three intramolecular hydrogen bonds involving the Glu NH_3_^+^ group, which interacts with the COOH, γ‐CO, and S^−^ functions of Glu and Cys, respectively. The C=O moiety of Gly remains free to interact with the metallic center at a distance of 3.41 Å (Figure 4). The other conformers of the cS family lie higher in energy and present similar H‐bond patterns; their structures are shown in Figure S12.

When the proton is not relocated from the thiol to the amino function, such as in the cSH isomer, the relative free energy increases significantly by up to 35.2 kJ mol^−1^ (**cSH-1**) or more, as reported in Figure S13, effectively ruling out its presence in the sampled ion population.

In the **eN-1** structure, which possesses almost the same free energy as **cS-1** (ΔG = 0.1 kJ mol^−1^), GSH coordinates the metal center via the α‐NH_2_ function of N‐terminal Glu. Here, the Cys side chain containing the SH moiety is free to establish hydrogen bonds with the NH group of Gly. The intramolecular H‐bond pattern is completed by interactions between each H atom of the coordinated NH_2_ group and the CO groups of Cys and Glu. Other eN conformers are characterized by comparable hydrogen bond patterns and are displayed in Figure S14.

The two free α‐carboxyl functions, belonging to Gly and Glu, can also coordinate the metallic center. Computational results indicate that the most stable isomers of these complexes are the corresponding zwitterionic forms with carboxylate and ammonium functions, **eCOO-1** and **gCOO-1**, with relative free energies of 16.3 and 48.7 kJ mol^−1^, respectively. This finding suggests that coordination via the Glu COO^−^ function is favored over that of Gly, likely because the positively charged Glu NH_3_^+^ function is located very close to the Au(I) center in **gCOO-1**. The lowest‐energy structures for eCOOH/eCOO and gCOOH/gCOO are shown in Figures S15 and S16, respectively, showing their distinct H‐bond patterns.

Considering their relatively high free energy values, the presence of gCOOH/gCOO and eCOOH isomers in the ion population is unlikely under the present experimental conditions, with the possible exception of **eCOO-1** in trace amounts.

In principle, the C=O function of the peptide bonds might also act as a nucleophilic center to coordinate Au(I). To explore the stability of this coordination type, we studied the cCO isomers, where the carbonyl function of Cys (involved in an amide bond) binds directly to the metal center. The most stable structures are shown in Figure S17. As expected, **cCO-1** exhibits high relative free energy (42.8 kJ mol^−1^), suggesting a negligible presence of isomers with an (NH)C=O–Au bond in the ion population.

For all optimized structures, we calculated IR spectra to help decipher the experimental IRMPD signatures. The IRMPD spectrum reported in Figure 5 shows the most intense features at 1785, 1520, and 1143 cm^−1^. The spectrum appears quite different from the one reported by Nieuwjaer et al. for [Au(GS‐H)]^-^ [18], in which features are present in the region between 1600 and 1700 cm^−1^, which agree with the presence of both a carboxylic acid and a carboxylate anionic group, and a strong absorption is reported at ca. 1350 cm^−1^. Photofragmentation spectra recorded on‐resonance at 1780 and 1140 cm^−1^, alongside the off‐resonance mass spectrum acquired at 900 cm^−1^, are presented in Figure S18 and display fragmentation patterns consistent with those observed under CID conditions. Based on their relative free energies, we expect the most abundant species to be **cS-1** and **eN-1**. A comparison of their theoretical spectra with experimental signatures (Figure 5) indicates that both isomers reproduce satisfactorily most of the spectrum, as detailed in Tables S3 and S4. Specifically, the absorptions at 1785, 1686, and 1143 cm^−1^ are well described by both isomers and are assigned to the C=O stretching mode of free carboxylic functions (Gly and Glu), Amide I (C=O stretching modes of the peptide bond), and Amide III (N–H bending and N–CO stretching of the peptide bond), respectively.

**FIGURE 5:**
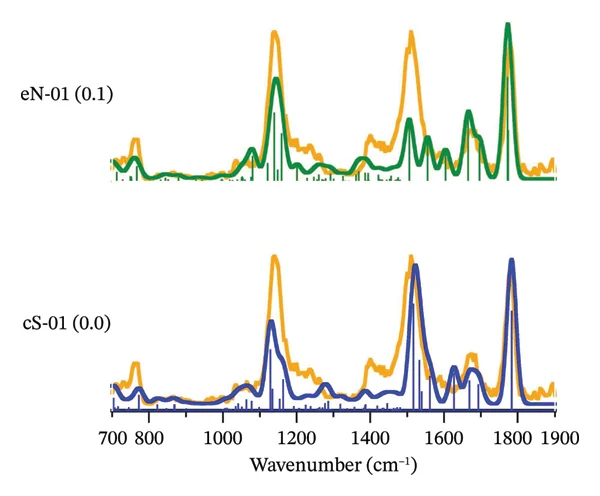
IRMPD spectrum (orange profiles) and calculated scaled harmonic IR spectra of the lowest energy isomers of the [(Et_3_P)AuGSH]^+^ ion, **cS-1** (blue profile) and **eN-1** (green profile), computed at B3LYP‐D3(BJ)/BS1 level of theory. Free energy values relative to **cS-1** are reported in parentheses in kJ mol^−1^.

However, the pronounced band at 1520 cm^−1^—the typical Amide II band—is well described only by the **cS-1** theoretical spectrum. In this region, the **cS-1** spectrum also contains the NH_3_^+^ umbrella mode, which cannot appear in **eN-1** because the NH_2_ moiety is involved in gold coordination. Furthermore, other cS conformers, though higher in energy, still exhibit a good description of the experimental signatures, especially in this region, implying their partial contribution based on relative free energy (Figure S19).

The band associated with umbrella mode is generally highly diagnostic in metal complexes involving amino acids with NH_2_ and other nucleophilic groups interacting with metallic centers, as shown in our previous work [25, 48]. Nevertheless, an intense absorption is present at 1501 cm^−1^ in the **eN-2** conformer (Figure S20), which is 5.3 kJ mol^−1^ higher in energy. This is assigned to the C–N stretching mode of the peptide bond formed between Glu and Cys, with the NH involved in a hydrogen bond with the SH moiety.

Thus, a comparison of the theoretical spectra computed for cS and eN (accounting for conformers within a 5–6 kJ mol^−1^ range), alongside their relative free energy values, suggests that both isomers coexist in the ion population with comparable abundances. This outcome aligns with the EAD data, where the observation of diagnostic channels for both S‐ and N‐coordination is consistent with the presence of two distinct isomeric forms.

ESI source is a soft ionization technique [49] that transfers the ionic species already present in solution into the gas phase [50]. Under these conditions, the amount of energy provided to the ions during phase transfer is limited and generally insufficient to promote high‐energy barrier rearrangements of the ion structures. Thus, the ions typically remain kinetically trapped in their solution‐phase configurations [51, 52], although in some cases kinetic trapping can be overcome by annealing into a thermal distribution [53].

As a consequence, ligand isomerization reactions in metal complexes, which require the cleavage of a coordinate covalent bond and are therefore characterized by high energy barriers, are in principle disfavored in ESI standard conditions. Thus, assuming the gas‐phase ion population to be a reliable description of the solution phase—excluding potential in‐source rearrangements during the ESI process—we explored the stability of **cS-1** and **eN-1** also in water and methanol. We found that the **eN** isomer is only ∼1.9 kJ mol^−1^ higher in free energy than cS in both solvents. Considering a Boltzmann distribution at standard room temperature (*T* = 298.15 K), this energy difference corresponds to a relative abundance of 68% (cS) and 32% (eN), which is consistent with the IRMPD and EAD analyses.

Therefore, ruling out significant gas‐phase or in‐source interconversion, AF ligand exchange in the presence of GSH seemingly leads to both **cS** and **eN** isomers in methanol/water solution.

The comparison of the theoretical signatures computed for other isomers (e.g., cSH, cCO, gCOOH/gCOO, and eCOOH/eCOO) with the experimental IRMPD spectrum shows a poor agreement, suggesting their negligible contribution to the experimental signature and thus corroborating the relative free energy analysis (Figures S21–S24).

### 3.3. Possible Implication for Reactivity in Biological Systems

In summary, correlating IRMPD and EAD data with DFT calculations suggests that in environments with a dielectric constant lower than bulk water—such as the present water/methanol mixture—GSH coordinates the Au(I) center via the cysteine thiol or the N‐terminal NH_2_ moiety with comparable affinity. This finding validates earlier observations regarding the AF reaction with Cys [25]. As a consequence, in cellular compartments where the dielectric constant drops relative to water [54], the GSH–Au(I) complex may exist as a mixture of S‐bound and N‐bound isomers. The presence of the latter, which retains a free thiol, could indeed impact GSH biological reactivity.

Previously, the N–Au binding motif had only been observed in proteins lacking free Cys and His residues. Specifically, Ferraro et al. [26] resolved the X‐ray structure of an Au(I)–carbene adduct with thaumatin (PDB ID: 5JVX), revealing that the gold atom coordinates the N‐terminal α‐NH_2_ of alanine (Figure 2) and the amine side chains of lysine and arginine residues (Arg8, Lys49, Lys97, Lys106, and Arg175).

Building upon these observations, our findings suggest potential new applications of Au(I)‐based drugs and provide testable hypotheses that warrant future experimental validation. We hypothesize that, despite the strong thiophilicity of Au(I), the N‐terminal NH_2_ group can serve as a competitive target for metalation even if free Cys residues are present, potentially favored by a local chemical environment that exhibits a low dielectric constant. Thus, enzymes whose activity critically depends on the N‐terminus may represent a promising class of targets for future investigation. A notable example is the family of N‐terminal nucleophile (Ntn) hydrolases. These enzymes rely on an N‐terminal residue that acts as a nucleophile, generally activated by its own unprotonated α‐NH_2_ group [55].

A prominent member of this family is the 20S proteasome [56, 57], where Au(III)‐based complexes have already demonstrated effective inhibition [58]. Other notable Ntn‐hydrolases include Penicillin G acylase (PGA), glutamine phosphoribosylpyrophosphate (PRPP) amidotransferase (GPAT), and Taspase 1. While PGA is crucial for industrial pharmaceutical sciences [59], GPAT and Taspase 1 are important targets for oncology.

In GPAT, which drives the rate‐limiting step in *de novo* purine biosynthesis, the N‐terminal Cys1 utilizes its own free NH_2_ terminus as a general base to enhance the nucleophilicity of its thiol side chain (PDB ID: 1ECF) [60–62]. Recent studies highlight that GPAT (often termed PPAT) fuels the progression of thyroid [63], nasopharyngeal [64], and hepatocellular carcinomas [65, 66]. Similarly, the α–β proenzyme endopeptidase Taspase employs a partially shielded N‐terminal threonine (Thr234) for substrate cleavage critical for tumor survival and apoptosis evasion [67, 68]. Following proenzyme cleavage, the free α‐amino group is essential for the nucleophilic attack of the Thr234 hydroxyl group (Figure S25, PDB ID: 2A8J) [69]. Since Taspase 1 depletion halts cancer cell proliferation and triggers apoptosis, deploying Au(I) compounds to block the α‐NH_2_ of this critical Thr residue represents an intriguing testable hypothesis for future therapeutic development.

## 4. Conclusion

In this work, we investigated the complex formed by AF and GSH, [(Et_3_P)AuGSH]^+^, by integrating IRMPD spectroscopy and EAD experimental data with theoretical calculations. We showed that in environments with a reduced dielectric constant, such as a methanol/water solution or the gas phase, the N‐bound isomer is present alongside the S‐bound form in comparable abundance. This finding might have important implications for GSH functionality in cellular regions characterized by a low dielectric constant. Moreover, it suggests a possible implication for the reactivity and potential applications of Au(I)‐based drugs: in the low‐dielectric environment of buried enzyme active sites, an unprotonated N‐terminal α‐NH_2_ group may provide a favorable site for metalation. Consequently, as a future direction, we hypothesize that small‐molecule Au(I) compounds could be explored as inhibitors of Ntn hydrolases implicated in cancer, such as GPAT and Taspase 1, through metalation of the N‐terminal α‐NH_2_ group that is essential for catalytic activation.

## Funding

The research leading to these results has received funding from LASERLAB‐EUROPE (Grant agreement no. 871124, European Union’s Horizon 2020 research and innovation program) and Sapienza Università di Roma (Grandi Attrezzature Scientifiche 2022, Grant no. GA122181AEFBE248; Progetti piccoli di Ateneo 2024, Grant no. RP1241910E5EA122). Open access publishing facilitated by Sapienza Università di Roma, as part of the Wiley‐CRUI‐CARE agreement.

## Disclosure

All authors read and approved the final manuscript.

## Conflicts of Interest

The authors declare no conflicts of interest.

## Supporting Information

Additional supporting information can be found online in the Supporting Information section.

## Supporting information


**Supporting Information** Figure S1: HR mass spectrum of the GSH–AF solution. Figure S2: CID mass spectrum of ${\left[\left({\text{Et}}_{3}\text{P}\right)\text{AuGSH}\right]}^{+}$. Figure S3: EAD mass spectrum of ${\left[\left({\text{Et}}_{3}\text{P}\right)\text{AuGSH}\right]}^{+}$. Figures S4–S6: Zoomed‐in mass spectral regions around m/z 401, 417, 461, and 475. Scheme S1: Proposed fragmentation pathway for the cS isomer. Scheme S2: Proposed fragmentation pathway for the eN isomer. Figures S7–S11: Molecular formula assignments and mass accuracy tolerance analyses for m/z 461, 417, 401, 621, and 475. Figures S12–S17: Optimized geometries of the lowest‐energy conformers for cS, cSH, eN, eCOOH/eCOO^−^, gCOOH/gCOO^−^, and cCO isomer families. Figure S18: Photofragmentation mass spectra recorded at 900, 1780, and 1140 cm^−1^. Figures S19–S24: Experimental IRMPD spectrum overlaid with calculated harmonic IR profiles for cS, eN, eCOOH/eCOO^−^, cSH, gCOOH/gCOO^−^, and cCO isomer families. Figure S25: Taspase 1 featuring the partially buried N‐terminal Thr234 adduct. Table S1: Molecular formulas and experimental mass errors for CID and EAD fragments. Table S2: Calculated relative free energies for ${\left[\left({\text{Et}}_{3}\text{P}\right)\text{AuGSH}\right]}^{+}$ isomers. Table S3: Experimental IRMPD absorptions and calculated vibrational frequencies for cS‐1. Table S4: Experimental IRMPD absorptions and calculated vibrational frequencies for eN‐1.

## Data Availability

The data that support the findings of this study are available in the Supporting Information of this article. The Cartesian coordinates of the optimized 3D structures that support the findings of this study are openly available in Zenodo at https://doi.org/10.5281/zenodo.22795472, reference number 70.

## References

[1] Yamashita M. , Auranofin: Past to Present, and Repurposing, International Immunopharmacology. (2021) 101, 10.1016/j.intimp.2021.108272. 34731781

[2] Martínez-González J. J. , Guevara-Flores A. , Álvarez G. , Rendón-Gómez J. L. , and del Arena I. P. , In Vitro Killing Action of Auranofin on Taenia crassiceps Metacestode (Cysticerci) and Inactivation of Thioredoxin–Glutathione Reductase (TGR), Parasitology Research. (2010) 107, no. 1, 227–231, 10.1007/s00436-010-1867-1. 20431894

[3] Zhang X. , Selvaraju K. , Ata Saei A. et al., Repurposing of Auranofin: Thioredoxin Reductase Remains a Primary Target of the Drug, Biochimie. (2019) 162, 46–54, 10.1016/j.biochi.2019.03.015. 30946948

[4] Zoppi C. , Messori L. , and Pratesi A. , ESI MS Studies Highligth the Selective Interaction of Auranofin With Protein Free Thiols, Dalton Transactions. (2020) 49, no. 18, 5906–5913, 10.1039/d0dt00283f. 32314767

[5] Griffith O. W. , Biologic and Pharmacologic Regulation of Mammalian Glutathione Synthesis, Free Radical Biology and Medicine. (1999) 27, no. 9-10, 922–935, 10.1016/s0891-5849(99)00176-8. 10569625

[6] Stephen Pearson A. and Cowan J. A. , Glutathione-Coordinated Metal Complexes as Substrates for Cellular Transporters, Metallomics. (2021) 13, no. 5, mfab015, 10.1093/mtomcs/mfab015. PMC8086996 33770183

[7] Antosova A. , Gazova Z. , Fedunova D. et al., Anti-Amyloidogenic Activity of Glutathione-Covered Gold Nanoparticles, Materials Science and Engineering: C. (2012) 32, no. 8, 2529–2535, 10.1016/j.msec.2012.07.036.

[8] Steckiewicz K. P. , Barcinska E. , Sobczak K. , Tomczyk E. , Wojcik M. , and Inkielewicz-Stepniak I. , Assessment of Anti-Tumor Potential and Safety of Application of Glutathione Stabilized Gold Nanoparticles Conjugated With Chemotherapeutics, International Journal of Medical Sciences. (2020) 17, no. 6, 824–833, 10.7150/ijms.40827. PMC7085271 32218704

[9] Habermann K. J. , Grünewald L. , van Wijk S. , and Fulda S. , Targeting Redox Homeostasis in Rhabdomyosarcoma Cells: Gsh-Depleting Agents Enhance Auranofin-Induced Cell Death, Cell Death and Disease. (2017) 8, no. 10, 10.1038/cddis.2017.412. PMC5680568 28981107

[10] You B. R. , Shin H. R. , Han B. R. , Kim S. H. , and Park W. H. , Auranofin Induces Apoptosis and Necrosis in Hela Cells via Oxidative Stress and Glutathione Depletion, Molecular Medicine Reports. (2015) 11, no. 2, 1428–1434, 10.3892/mmr.2014.2830. 25370167

[11] You B. R. and Park W. H. , Auranofin Induces Mesothelioma Cell Death Through Oxidative Stress and GSH Depletion, Oncology Reports. (2016) 35, no. 1, 546–551, 10.3892/or.2015.4382. 26530353

[12] Ghini V. , Senzacqua T. , Massai L. , Gamberi T. , Messori L. , and Turano P. , NMR Reveals the Metabolic Changes Induced by Auranofin in A2780 Cancer Cells: Evidence for Glutathione Dysregulation, Dalton Transactions. (2021) 50, no. 18, 6349–6355, 10.1039/d1dt00750e. 33885689

[13] Rigobello M. P. , Scutari G. , Boscolo R. , and Bindoli A. , Induction of Mitochondrial Permeability Transition by Auranofin, A Gold(I)-Phosphine Derivative, British Journal of Pharmacology. (2002) 136, no. 8, 1162–1168, 10.1038/sj.bjp.0704823. PMC1573451 12163349

[14] Roberts J. R. and Shaw C. F.III, Inhibition of Erythrocyte Selenium-Glutathione Peroxidase by Auranofin Analogues and Metabolites, Biochemical Pharmacology. (1998) 55, no. 8, 1291–1299, 10.1016/s0006-2952(97)00634-5. 9719485

[15] Chaudieáre J. and Tappel A. L. , Interaction of Gold(I) With the Active Site of Selenium-Glutathione Peroxidase, Journal of Inorganic Biochemistry. (1984) 20, 313–325. 10.1016/0162-0134(84)85030-8 6425459

[16] MacAleese L. and Maître P. , Mass Infrared Spectroscopy of Organometallic Ions in the Gas Phase: From Model to Real World Complexes, Mass Spectrometry Reviews. (2007) 26, no. 4, 583–605, 10.1002/mas.20138. 17471578

[17] Oomens J. , Polfer N. C. , Berden G. , and Eyler J. R. , Gas-Phase Metal Ion Chelation Investigated With IRMPD Spectroscopy: A Brief Review of Robert Dunbar’s Contributions, European Journal of Mass Spectrometry. (2019) 25, no. 1, 86–96, 10.1177/1469066718799175. 30205710

[18] Nieuwjaer N. , Beydoun A. , Lecomte F. , Manil B. , Scuderi D. , and Desfrançois C. , Ligand-Protected Gold Nanoclusters Probed by IRMPD Spectroscopy and Quantum Chemistry Calculations, Journal of Molecular Spectroscopy. (2022) 383, 10.1016/j.jms.2021.111562. 33353333

[19] Yamaguchi M. , Zhang Y. , Lushchikova O. V. , Bakker J. M. , and Mafuné F. , Structures of Nitrogen Oxides Attached to Anionic Gold Clusters Au_4_ ^−^ Revealed by Infrared Multiple Photon Dissociation Spectroscopy, Journal of Physical Chemistry A. (2021) 125, no. 41, 9040–9047, 10.1021/acs.jpca.1c05381. 34636578

[20] Škríba A. , Jâsíková L. , and Roithová J. , Silver(I) and Gold(I) Complexes of Diethylmalonate, International Journal of Mass Spectrometry. (2012) 330-332, 226–232, 10.1016/j.ijms.2012.08.019.

[21] Roithová J. , Janková Š. , Jašková L. , Váňa J. , and Hybelbauerová S. , Gold–Gold Cooperation in the Addition of Methanol to Alkynes, Angewandte Chemie. (2012) 124, 8503–8507. 10.1002/anie.201204003 22782688

[22] Gregori B. , Guidoni L. , Chiavarino B. et al., Vibrational Signatures of S-Nitrosoglutathione as Gaseous, Protonated Species, Journal of Physical Chemistry B. (2014) 118, no. 43, 12371–12382, 10.1021/jp5072742. 25274121

[23] Osburn S. , Berden G. , Oomens J. et al., Structure and Reactivity of the Glutathione Radical Cation: Radical Rearrangement From the Cysteine Sulfur to the Glutamic Acid A-Carbon Atom, ChemPlusChem. (2013) 78, no. 9, 970–978, 10.1002/cplu.201300057. 31986733

[24] Walker S. K. , Bubas A. R. , Stevenson B. C. et al., Structural Determination of Zn^2+^, Cu^2+^, and Fe^2+^ Complexed With Glutathione by IRMPD Spectroscopy and Complimentary Ab Initio Calculations, Physical Chemistry Chemical Physics. (2024) 26, no. 47, 29406–29419, 10.1039/d4cp03848g. 39575572

[25] Paciotti R. , Corinti D. , Coletti C. et al., Vibrational Analysis of Auranofin Complexes With Cysteine and Selenocysteine Unveils Distinct Binding Motifs and Specific Unimolecular Reactivity, Inorganic Chemistry Frontiers. (2024) 11, no. 23, 8464–8478, 10.1039/d4qi02023e.

[26] Ferraro G. , Gabbiani C. , and Merlino A. , First Crystal Structure for a Gold Carbene−Protein Adduct, Bioconjugate Chemistry. (2016) 27, no. 7, 1584–1587, 10.1021/acs.bioconjchem.6b00298. 27364343

[27] Tolbatov I. , Marrone A. , Coletti C. , and Re N. , Computational Studies of Au(I) and Au(III) Anticancer Metallodrugs: A Survey, Molecules. (2021) 26, no. 24, 10.3390/molecules26247600. PMC8707411 34946684

[28] Loboda A. V. and Chernushevich I. V. , A Novel Ion Trap for Duty Cycle Improvement in an Orthogonal-Acceleration TOF Mass Spectrometer, Journal of the American Society for Mass Spectrometry. (2009) 20, 773–784. 10.1016/j.jasms.2009.03.018 19394860

[29] Oepts D. , van der Meer A. F. G. , and van Amersfoort P. W. , The Free-Electron-Laser User Facility FELIX, Infrared Physics and Technology. (1995) 36, no. 1, 297–308, 10.1016/1350-4495(94)00074-u.

[30] Martens J. , Berden G. , Gebhardt C. R. , and Oomens J. , Infrared Ion Spectroscopy in a Modified Quadrupole Ion Trap Mass Spectrometer at the FELIX Free Electron Laser Laboratory, Review of Scientific Instruments. (2016) 87, no. 10, 10.1063/1.4964703. 27802712

[31] Berden G. , Derksen M. , Houthuijs K. J. , Martens J. , and Oomens J. , An Automatic Variable Laser Attenuator for IRMPD Spectroscopy and Analysis of Power-Dependence in Fragmentation Spectra, International Journal of Mass Spectrometry. (2019) 443, 1–8, 10.1016/j.ijms.2019.05.013.

[32] Prell J. S. , O’Brien J. T. , and Williams E. R. , IRPD Spectroscopy and Ensemble Measurements: Effects of Different Data Acquisition and Analysis Methods, Journal of the American Society for Mass Spectrometry. (2010) 21, no. 5, 800–809, 10.1016/j.jasms.2010.01.010. 20185332

[33] van Geenen F. A. M. G. , Kranenburg R. F. , van Asten A. C. , Martens J. , Oomens J. , and Berden G. , Isomer-Specific Two-Color Double-Resonance IR^2^MS^3^ Ion Spectroscopy Using a Single Laser: Application in the Identification of Novel Psychoactive Substances, Analytical Chemistry. (2021) 93, no. 4, 2687–2693, 10.1021/acs.analchem.0c05042. PMC7859929 33470107

[34] Bannwarth C. , Ehlert S. , and Grimme S. , GFN2-xTB—An Accurate and Broadly Parametrized Self-Consistent Tightbinding Quantum Chemical Method With Multipole Electrostatics and Density-Dependent Dispersion Contributions, Journal of Chemical Theory and Computation. (2019) 15, no. 3, 1652–1671, 10.1021/acs.jctc.8b01176. 30741547

[35] Bannwarth C. , Caldeweyher E. , Ehlert S. et al., Extended Tight-Binding Quantum Chemistry Methods, Wiley Interdisciplinary Reviews: Computational Molecular Science. (2021) 11, no. 2, 10.1002/wcms.1493.

[36] Pracht P. , Bohle F. , and Grimme S. , Automated Exploration of the Low-Energy Chemical Space With Fast Quantum Chemical Methods, Physical Chemistry Chemical Physics. (2020) 22, no. 14, 7169–7192, 10.1039/c9cp06869d. 32073075

[37] Pracht P. , Grimme S. , Bannwarth C. et al., CREST—A Program for the Exploration of Low-Energy Molecular Chemical Space, Journal of Chemical Physics. (2024) 160, no. 11, 10.1063/5.0197592. 38511658

[38] Grimme S. , Ehrlich S. , and Goerigk L. , Effect of the Damping Function in Dispersion Corrected Density Functional Theory, Journal of Computational Chemistry. (2011) 32, no. 7, 1456–1465, 10.1002/jcc.21759. 21370243

[39] Pritchard B. P. , Altarawy D. , Didier B. , Gibson T. D. , and Windus T. L. , New Basis Set Exchange: An Open, Up-to-Date Resource for the Molecular Sciences Community, Journal of Chemical Information and Modeling. (2019) 59, no. 11, 4814–4820, 10.1021/acs.jcim.9b00725. 31600445

[40] Onsager L. , Electric Moments of Molecules in Liquids, Journal of the American Chemical Society. (1936) 58, no. 8, 1486–1493, 10.1021/ja01299a050.

[41] Barone V. and Cossi M. , Quantum Calculation of Molecular Energies and Energy Gradients in Solution by a Conductor Solvent Model, Journal of Physical Chemistry A. (1998) 102, no. 11, 1995–2001, 10.1021/jp9716997.

[42] Cossi M. , Rega N. , Scalmani G. , and Barone V. , Energies, Structures, and Electronic Properties of Molecules in Solution With the C–PCM Solvation Model, Journal of Computational Chemistry. (2003) 24, no. 6, 669–681, 10.1002/jcc.10189. 12666158

[43] Marenich A. V. , Cramer C. J. , and Truhlar D. G. , Universal Solvation Model Based on Solute Electron Density and on a Continuum Model of the Solvent Defined by the Bulk Dielectric Constant and Atomic Surface Tensions, Journal of Physical Chemistry B. (2009) 113, no. 18, 6378–6396, 10.1021/jp810292n. 19366259

[44] Andreussi O. , Hörmann N. G. , Nattino F. , Fisicaro G. , Goedecker S. , and Marzari N. , Solvent-Aware Interfaces in Continuum Solvation, Journal of Chemical Theory and Computation. (2019) 15, no. 3, 1996–2009, 10.1021/acs.jctc.8b01174. 30682250

[45] Frisch M. J. , Trucks G. W. , Schlegel H. B. et al., Gaussian 09, Revision D.01, 2009, Gaussian Inc.

[46] Shoeib T. , Atkinson D. W. , and Sharp B. L. , Structural Analysis of the Anti-Arthritic Drug Auranofin: Its Complexes With Cysteine, Selenocysteine and Their Fragmentation Products, Inorganica Chimica Acta. (2010) 363, no. 1, 184–192, 10.1016/j.ica.2009.08.034.

[47] Bons J. , Hunter C. L. , Chupalov R. et al., Localization and Quantification of Post-Translational Modifications of Proteins Using Electron Activated Dissociation Fragmentation on a Fast-Acquisition Time-of-Flight Mass Spectrometer, Journal of the American Society for Mass Spectrometry. (2023) 34, no. 10, 2199–2210, 10.1021/jasms.3c00144. PMC11157679 37694881

[48] Paciotti R. , Corinti D. , De Petris A. et al., Cisplatin and Transplatin Interaction With Methionine: Bonding Motifs Assayed by Vibrational Spectroscopy in the Isolated Ionic Complexes, Physical Chemistry Chemical Physics. (2017) 19, no. 39, 26697–26707, 10.1039/c7cp05203k. 28876340

[49] Bhardwaj C. and Hanley L. , Ion Sources for Mass Spectrometric Identification and Imaging of Molecular Species, Natural Product Reports. (2014) 31, no. 6, 756–767, 10.1039/c3np70094a. 24473154

[50] Ho C. S. , Lam C. W. K. , Chan M. H. M. et al., Electrospray Ionisation Mass Spectrometry: Principles and Clinical Applications, Clinical Biochemist Reviews. (2003) 24, no. 1, 3–12. PMC1853331 18568044

[51] Zhu Y. , Roy H. A. , Cunningham N. A. et al., IRMPD Action Spectroscopy, ER-CID Experiments, and Theoretical Studies of Sodium Cationized Thymidine and 5-Methyluridine: Kinetic Trapping During the ESI Desolvation Process Preserves the Solution Structure of [Thd+Na]^+^ , Journal of the American Society for Mass Spectrometry. (2017) 28, no. 11, 2423–2437, 10.1007/s13361-017-1753-5. 28836109

[52] Steill J. D. and Oomens J. , Gas-Phase Deprotonation of p-Hydroxybenzoic Acid Investigated by IR Spectroscopy: Solution-Phase Structure is Retained Upon ESI, Journal of the American Chemical Society. (2009) 131, no. 38, 13570–13571, 10.1021/ja903877v. 19731908

[53] Prell J. S. , Chang T. M. , Biles J. A. , Berden G. , Oomens J. , and Williams E. R. , Isomer Population Analysis of Gaseous Ions From Infrared Multiple Photon Dissociation Kinetics, Journal of Physical Chemistry A. (2011) 115, no. 13, 2745–2751, 10.1021/jp2004166. 21410158

[54] Sakota K. , Tabata D. , and Sekiya H. , Macromolecular Crowding Modifies the Impact of Specific Hofmeister Ions on the Coil–Globule Transition of PNIPAM, Journal of Physical Chemistry B. (2015) 119, no. 32, 10334–10340, 10.1021/acs.jpcb.5b01255. 26215482

[55] Brannigan J. A. , Dodson G. , Duggleby H. J. et al., A Protein Catalytic Framework With an N-Terminal Nucleophile is Capable of Self-Activation, Nature. (1995) 378, no. 6555, 416–419, 10.1038/378416a0. 7477383

[56] Groll M. , Ditzel L. , Löwe J. et al., Structure of 20S Proteasome From Yeast at 2.4 Å Resolution, Nature. (1997) 386, no. 6624, 463–471, 10.1038/386463a0. 9087403

[57] Huber E. M. , Heinemeyer W. , Li X. , Arendt C. S. , Hochstrasser M. , and Groll M. , A Unified Mechanism for Proteolysis and Autocatalytic Activation in the 20S Proteasome, Nature Communications. (2016) 7, no. 1, 10.1038/ncomms10900. PMC4792962 26964885

[58] Milacic V. and Dou Q. P. , The Tumor Proteasome as a Novel Target for Gold(III) Complexes: Implications for Breast Cancer Therapy, Coordination Chemistry Reviews. (2009) 253, no. 11-12, 1649–1660, 10.1016/j.ccr.2009.01.032. PMC2675785 20047011

[59] Roopa L. and Shanthi V. , Penicillin G Acylase: A Multifunctional Catalyst With Broad Implications in Pharmaceutical Science and Industrial Applications, Letters in Drug Design and Discovery. (2025) 21, 4683–4697.

[60] Kim J. H. , Krahn J. M. , Tomchick D. R. , Smith J. L. , and Zalkin H. , Structure and Function of the Glutamine Phosphoribosylpyrophosphate Amidotransferase Glutamine Site and Communication With the Phosphoribosylpyrophosphate Site, Journal of Biological Chemistry. (1996) 271, no. 26, 15549–15557, 10.1074/jbc.271.26.15549. 8663035

[61] Isupov M. N. , Obmolova G. , Butterworth S. et al., Substrate Binding is Required for Assembly of the Active Conformation of the Catalytic Site in Ntn Amidotransferases: Evidence From the 1.8 Å Crystal Structure of the Glutaminase Domain of Glucosamine 6-Phosphate Synthase, Structure. (1996) 4, no. 7, 801–810, 10.1016/s0969-2126(96)00087-1. 8805567

[62] Muchmore C. R. , Krahn J. M. , Smith J. L. , Kim J. H. , and Zalkin H. , Crystal Structure of Glutamine Phosphoribosylpyrophosphate Amidotransferase From *Escherichia coli* , Protein Science. (1998) 7, no. 1, 39–51, 10.1002/pro.5560070104. PMC2143822 9514258

[63] Liu B. , Song M. , Qin H. et al., Phosphoribosyl Pyrophosphate Amidotransferase Promotes the Progression of Thyroid Cancer via Regulating Pyruvate Kinase M2, OncoTargets and Therapy. (2020) 13, 7629–7639, 10.2147/ott.s253137. PMC7413720 32801776

[64] Kitagawa Y. , Kondo S. , Fukuyo M. et al., Phosphoribosyl Pyrophosphate Amidotransferase: Novel Biomarker and Therapeutic Target for Nasopharyngeal Carcinoma, Cancer Science. (2024) 115, no. 11, 3587–3595, 10.1111/cas.16314. PMC11531959 39196700

[65] Zhou J. , Dong C. , Xu S. et al., ppAT Drives Glutamine-Dependent Purine Biosynthesis and Malignant Progression in Hepatocellular Carcinoma, BMC Cancer. (2026) 26, no. 1, 10.1186/s12885-025-15512-y. PMC12911291 41559600

[66] Chu Q. , Gu X. , Zheng Q. , Wang J. , and Zhu H. , Phosphoribosyl Pyrophosphate Amido Transferase: A New Prognostic Biomarker for Hepatocellular Carcinoma, International Journal of General Medicine. (2022) 15, 353–358, 10.2147/ijgm.s340758. PMC8752061 35027843

[67] Hsieh J. J. D. , Cheng E. H. Y. , and Korsmeyer S. J. , Taspase1: A Threonine Aspartase Required for Cleavage of MLL and Proper HOX Gene Expression, Cell. (2003) 115, no. 3, 293–303, 10.1016/s0092-8674(03)00816-x. 14636557

[68] Thornberry N. A. and Lazebnik Y. , Caspases: Enemies Within, Science. (1998) 281, no. 5381, 1312–1316, 10.1126/science.281.5381.1312. 9721091

[69] Khan J. A. , Dunn B. M. , and Tong L. , Crystal Structure of Human Taspase1, A Crucial Protease Regulating the Function of MLL, Structure. (2005) 13, no. 10, 1443–1452, 10.1016/j.str.2005.07.006. 16216576

