# Supplementary material for: Reevaluating Gold(I) Thiophilicity in Reduced Dielectric Environments: IRMPD Spectroscopy and Theoretical Investigation of Auranofin–Glutathione Complexes
Source: Bioinorg Chem Appl. 2026 Oct 19;2026:5944632. doi: 10.1155/bca/5944632 (PMC13680860; doi:10.1155/bca/5944632)
Supplement: Supplementary file 1 [file BCA-2026-5944632-s001.docx]

***Supporting Information***

**Reevaluating Gold(I) Thiophilicity in Reduced Dielectric Environments: IRMPD Spectroscopy and Theoretical Investigation of Auranofin-Glutathione Complexes**

*Roberto Paciotti,*^a^ Susanna Grasso,^b^ Cecilia Coletti,^a^ Nazzareno Re,^a^ Giel Berden,^c,d^ Jos Oomens,^c,d^ Charles Desfrançois, ^e^ Simonetta Fornarini,^b^ Davide Corinti,*^b^ Maria Elisa Crestoni^b^*

^a^ *Dipartimento di Farmacia, Università G. D’Annunzio Chieti-Pescara, Via dei Vestini 31, Chieti I-66100, Italy.*

^b^ *Dipartimento di Chimica e Tecnologie del Farmaco, Università di Roma “La Sapienza”, I-00185 Roma, Italy.*

*^c^ HFML-FELIX, Toernooiveld 7, 6525ED Nijmegen, the Netherlands*

*^d^ Institute for Molecules and Materials, Radboud University, Heyendaalseweg 135, 6525AJ Nijmegen, the Netherlands.*

*^e^ Université Sorbonne Paris Nord, Lab. de Physique des Lasers, CNRS, 93430 Villetaneuse, France.*

**Correspondence:** Roberto Paciotti (r.paciotti@unich.it) | Davide Corinti (davide.corinti@uniroma1.it)

**Index**

**Figure S1:** HR mass spectrum (ZenoTOF7600) of the GSH-auranofin water/methanol solution…….4

**Figure S2:** CID mass spectrum (CE = 20 V) of [(Et_3_P)AuGSH]^+^ at *m/z* 622…...…………………......5

**Figure S3:** EAD mass spectrum (CE = 10 V, Electron KE = 10 eV) of [(Et_3_P)AuGSH]^+^ at *m/z* 622…6

**Figure S4:** Zoomed-in view of the region around m/z 401.0389 of the CID (top) and EAD (bottom) mass spectra ……………………………………………………………………………………………7

**Figure S5:** Zoomed-in view of the region around m/z 417.1352 of the CID (top) and EAD (bottom) mass spectra…………………………………………………………………………………………….7

**Figure S6:** Zoomed-in view of the region around m/z 461.1257 and 475.0631 of the CID (top) and EAD (bottom) mass spectra…………………………………………………………………………….8

**Scheme S1:** Proposed fragmentation pathway for the **cS** isomer of [(Et_3_P)AuGSH]^+^…………………9

**Scheme S2:** Proposed fragmentation pathway for the **eN** isomer of [(Et_3_P)AuGSH]^+^……………….10

**Figure S7:** Molecular formula assignment of m/z 461.1258 by elemental composition analysis using a mass tolerance of 10 ppm……………………………………………………………………………...11

**Figure S8:** Molecular formula assignment of m/z 417.1353 by elemental composition analysis using a mass tolerance of 10 ppm. ……………………….…………………………………………………....11

**Figure S9:** Molecular formula assignment of m/z 401.0391 by elemental composition analysis using a mass tolerance of 10 ppm. …………………………………………………………………………….11

**Figure S10:** Molecular formula assignment of m/z 621.1297 by elemental composition analysis using a mass tolerance of 10 ppm. …………………………………………………………………………..11

**Figure S11:** Molecular formula assignment of m/z 475.0632 by elemental composition analysis using a mass tolerance of 10 ppm. ………………………………………………………………………......12

**Figure S12:** Optimized geometries of the lowest energy conformers of the **cS** isomer family, computed in gas phase at B3LYP-D3(BJ)/BS1 level of theory. Free energy values relative to **cS-1** are reported in parenthesis in kJ mol^−1^. Hydrogen bond distances (Å) are indicated by red dotted lines……………………………………………………………………………………………………13

**Figure S13:** Optimized geometries of the lowest energy conformers of the **cSH** isomer family, computed in gas phase at B3LYP-D3(BJ)/BS1 level of theory. Free energy values relative to **cS-1** are reported in parenthesis in kJ mol^−1^. Hydrogen bond distances (Å) are indicated by red dotted lines……………………………………………………………………………………………………14

**Figure S14:** Optimized geometries of the lowest energy conformers of the **eN** isomer family, computed in gas phase at B3LYP-D3(BJ)/BS1 level of theory. Free energy values relative to **cS-1** are reported in parenthesis in kJ mol^−1^. Hydrogen bond distances (Å) are indicated by red dotted lines……………………………………………………………………………………………………15

**Figure S15:** Optimized geometries of the lowest energy conformers of the **eCOOH**/**eCOO** isomer family, computed in gas phase at B3LYP-D3(BJ)/BS1 level of theory. Free energy values relative to **cS-1** are reported in parenthesis in kJ mol^−1^. Hydrogen bond distances (Å) are indicated by red dotted lines……………………………………………………………………………………………………16

**Figure S16:** Optimized geometries of the lowest energy conformers of the **gCOOH**/**gCOO** isomer family, computed in gas phase at B3LYP-D3(BJ)/BS1 level of theory. Free energy values relative to **cS-1** are reported in parenthesis in kJ mol^−1^. Hydrogen bond distances (Å) are indicated by red dotted lines……………………………………………………………………………………………………17

**Figure S17:** Optimized geometries of the lowest energy conformers of the **cCO** isomer family, computed in gas phase at B3LYP-D3(BJ)/BS1 level of theory. Free energy values relative to **cS-1** are reported in parenthesis in kJ mol^−1^. Hydrogen bond distances (Å) are indicated by red dotted lines........................................................................................................................................................18

**Figure S18:** Photofragmentation mass spectra of [(Et_3_P)AuGSH]^+^ recorded with the FELIX FEL tuned at 900, 1780 and 1140 cm^−1^. The fragment at *m/z* 333, attributable to [(Et_3_P)AuH_2_O]^+^ is due to the presence of trace water in the ion trap…………………………………………………………….19

**Figure S19:** IRMPD spectrum (orange profiles) and calculated harmonic IR spectra of the lowest energy structures of **cS** isomer family (blue profiles), computed in gas phase at B3LYP-D3(BJ)/BS1 level of theory. Free energy values relative to **cS-1** are reported in parenthesis in kJ mol^−1^. Harmonic frequencies were scaled by 0.98………………………………………………………………………20

**Figure S20:** IRMPD spectrum (orange profiles) and calculated harmonic IR spectra of the lowest energy structures of **eN** isomer family (blue profiles), computed in gas phase at B3LYP-D3(BJ)/BS1 level of theory. Free energy values relative to **cS-1** are reported in parenthesis in kJ mol^−1^. Harmonic frequencies were scaled by 0.98………………………………………………………………………21

**Figure S21:** IRMPD spectrum (orange profiles) and calculated harmonic IR spectra of the lowest energy structures of **eCOOH**/**eCOO** isomer family (black profiles), computed in gas phase at B3LYP-D3(BJ)/BS1 level of theory. Free energy values relative to **cS-1** are reported in parenthesis in kJ mol^−1^. Harmonic frequencies were scaled by 0.98………………………………………………....22

**Figure S22:** IRMPD spectrum (orange profiles) and calculated harmonic IR spectra of the lowest energy structures of **cSH** isomer family (black profiles), computed in gas phase at B3LYP-D3(BJ)/BS1 level of theory. Free energy values relative to **cS-1** are reported in parenthesis in kJ mol^−1^. Harmonic frequencies were scaled by 0.98.........................................................................................................................................................23

**Figure S23:** IRMPD spectrum (orange profiles) and calculated harmonic IR spectra of the lowest energy structures of **gCOOH**/**gCOO** isomer family (black profiles), computed in gas phase at B3LYP-D3(BJ)/BS1 level of theory. Free energy values relative to **cS-1** are reported in parenthesis in kJ mol^−1^. Harmonic frequencies were scaled by 0.98………………………………………………....24

**Figure S24:** IRMPD spectrum (orange profiles) and calculated harmonic IR spectra of the lowest energy structures of **cCO** isomer family (black profiles), computed in gas phase at B3LYP-D3(BJ)/BS1 level of theory. Free energy values relative to **cS-1** are reported in parenthesis in kJ mol^−1^. Harmonic frequencies were scaled by 0.98……………………………………………………25

**Figure S25:** Taspase 1 featuring the partially buried N-terminal Thr234 adduct (adapted from PDB ID: 2A8J [66]).; the unprotonated α-NH_2_ group acts as a general base in the catalytic mechanism and may serve as a target for Au(I) complexes. The carbon, nitrogen and oxygen atoms of Thr234 are shown in gray, blue and red, respectively, while the protein’s molecular surface in light blue………26

**Table S1:** Molecular formula attributed to CID and EAD fragments from [(Et_3_P)AuGSH]^+^ together with the error in ppm between calculated exact mass and reported experimental *m/z*………………..27

**Table S2:** free energies values (kJ mol^-1^) of isomers and conformers of [(Et_3_P)AuGSH]^+^ ion relative to **cS-1**, computed in the gas phase at the B3LYP-D3(BJ)/BS1 level of theory………………………………………………………………………………………………….28

**Table S3:** IRMPD absorptions of [(Et_3_P)AuGSH]^+^ ion and calculated vibrational frequencies (cm^-1^) for **cS-1**. Calculated intensities are reported in parentheses (km mol^-1^). Calculated frequencies in the fingerprint (700-1900 cm^-1^) and range are scaled by factors of 0.98. Non relevant modes with intensity lower than 40 km mol^-1^ are omitted…………………………………………………………………...29

**Table S4:** IRMPD absorptions of [(Et_3_P)AuGSH]^+^ ion and calculated vibrational frequencies (cm^-1^) for **eN-1**. Calculated intensities are reported in parentheses (km mol^-1^). Calculated frequencies in the fingerprint (700-1900 cm^-1^) and range are scaled by factors of 0.98. Non relevant modes with intensity lower than 40 km mol^-1^ are omitted…………………………………………………………………...30

**Figure S1:** HR mass spectrum (ZenoTOF7600) of the GSH-auranofin water/methanol solution.

**Figure S2:** CID mass spectrum (CE = 20 V) of [(Et_3_P)AuGSH]^+^ at *m/z* 622.

475.0615

401.0371

417.1365

**Figure S3:** EAD mass spectrum (CE = 10 V, Electron KE = 10 eV) of [(Et_3_P)AuGSH]^+^ at *m/z* 622.

**Figure S4:** Zoomed-in view of the region around m/z 401.0389 of the CID (top) and EAD (bottom) mass spectra

**Figure S5:** Zoomed-in view of the region around m/z 417.1352 of the CID (top) and EAD (bottom) mass spectra

**Figure S6:** Zoomed-in view of the region around m/z 461.1257 and 475.0631 of the CID (top) and EAD (bottom) mass spectra

**Scheme S1:** Proposed fragmentation pathway for the **cS** isomer of [(Et_3_P)AuGSH]^+^.

**Scheme S2:** Proposed fragmentation pathway for the **eN** isomer of [(Et_3_P)AuGSH]^+^.

**Figure S7:** Molecular formula assignment of m/z 461.1258 by elemental composition analysis using a mass tolerance of 10 ppm.

**Figure S8:** Molecular formula assignment of m/z 417.1353 by elemental composition analysis using a mass tolerance of 10 ppm.

**Figure S9:** Molecular formula assignment of m/z 401.0391 by elemental composition analysis using a mass tolerance of 10 ppm.

**Figure S10:** Molecular formula assignment of m/z 621.1297 by elemental composition analysis using a mass tolerance of 10 ppm.

**Figure S11:** Molecular formula assignment of m/z 475.0632 by elemental composition analysis using a mass tolerance of 10 ppm.

**Figure S12:** Optimized geometries of the lowest energy conformers of the **cS** isomer family, computed in gas phase at B3LYP-D3(BJ)/BS1 level of theory. Free energy values relative to **cS-1** are reported in parenthesis in kJ mol^−1^. Hydrogen bond distances (Å) are indicated by red dotted lines.

**Figure S13:** Optimized geometries of the lowest energy conformers of the **cSH** isomer family, computed in gas phase at B3LYP-D3(BJ)/BS1 level of theory. Free energy values relative to **cS-1** are reported in parenthesis in kJ mol^−1^. Hydrogen bond distances (Å) are indicated by red dotted lines.

**Figure S14:** Optimized geometries of the lowest energy conformers of the **eN** isomer family, computed in gas phase at B3LYP-D3(BJ)/BS1 level of theory. Free energy values relative to **cS-1** are reported in parenthesis in kJ mol^−1^. Hydrogen bond distances (Å) are indicated by red dotted lines.

**Figure S15:** Optimized geometries of the lowest energy conformers of the **eCOOH**/**eCOO** isomer family, computed in gas phase at B3LYP-D3(BJ)/BS1 level of theory. Free energy values relative to **cS-1** are reported in parenthesis in kJ mol^−1^. Hydrogen bond distances (Å) are indicated by red dotted lines.

**Figure S16:** Optimized geometries of the lowest energy conformers of the **gCOOH**/**gCOO** isomer family, computed in gas phase at B3LYP-D3(BJ)/BS1 level of theory. Free energy values relative to **cS-1** are reported in parenthesis in kJ mol^−1^. Hydrogen bond distances (Å) are indicated by red dotted lines.

**Figure S17:** Optimized geometries of the lowest energy conformers of the **cCO** isomer family, computed in gas phase at B3LYP-D3(BJ)/BS1 level of theory. Free energy values relative to **cS-1** are reported in parenthesis in kJ mol^−1^. Hydrogen bond distances (Å) are indicated by red dotted lines.

**Figure S18:** Photofragmentation mass spectra of [(Et_3_P)AuGSH]^+^ recorded with the FELIX FEL tuned at 900, 1780 and 1140 cm^−1^. The fragment at *m/z* 333, attributable to [(Et_3_P)AuH_2_O]^+^ is due to the presence of trace water in the ion trap

**Figure S19:** IRMPD spectrum (orange profiles) and calculated harmonic IR spectra of the lowest energy structures of **cS** isomer family (blue profiles), computed in gas phase at B3LYP-D3(BJ)/BS1 level of theory. Free energy values relative to **cS-1** are reported in parenthesis in kJ mol^−1^. Harmonic frequencies were scaled by 0.98.

**Figure S20:** IRMPD spectrum (orange profiles) and calculated harmonic IR spectra of the lowest energy structures of **eN** isomer family (blue profiles), computed in gas phase at B3LYP-D3(BJ)/BS1 level of theory. Free energy values relative to **cS-1** are reported in parenthesis in kJ mol^−1^. Harmonic frequencies were scaled by 0.98.

**Figure S21:** IRMPD spectrum (orange profiles) and calculated harmonic IR spectra of the lowest energy structures of **eCOOH**/**eCOO** isomer family (black profiles), computed in gas phase at B3LYP-D3(BJ)/BS1 level of theory. Free energy values relative to **cS-1** are reported in parenthesis in kJ mol^−1^. Harmonic frequencies were scaled by 0.98.

**Figure S22:** IRMPD spectrum (orange profiles) and calculated harmonic IR spectra of the lowest energy structures of **cSH** isomer family (black profiles), computed in gas phase at B3LYP-D3(BJ)/BS1 level of theory. Free energy values relative to **cS-1** are reported in parenthesis in kJ mol^−1^. Harmonic frequencies were scaled by 0.98.

**Figure S23:** IRMPD spectrum (orange profiles) and calculated harmonic IR spectra of the lowest energy structures of **gCOOH**/**gCOO** isomer family (black profiles), computed in gas phase at B3LYP-D3(BJ)/BS1 level of theory. Free energy values relative to **cS-1** are reported in parenthesis in kJ mol^−1^. Harmonic frequencies were scaled by 0.98.

**Figure S24:** IRMPD spectrum (orange profiles) and calculated harmonic IR spectra of the lowest energy structures of **cCO** isomer family (black profiles), computed in gas phase at B3LYP-D3(BJ)/BS1 level of theory. Free energy values relative to **cS-1** are reported in parenthesis in kJ mol^−1^. Harmonic frequencies were scaled by 0.98.

**Figure S25:** Taspase 1 featuring the partially buried N-terminal Thr234 adduct (adapted from PDB ID: 2A8J [66]); the unprotonated α-NH_2_ group acts as a general base in the catalytic mechanism and may serve as a target for Au(I) complexes. The carbon, nitrogen and oxygen atoms of Thr234 are shown in gray, blue and red, respectively, while the protein’s molecular surface in light blue.

**Table S1:** Molecular formula attributed to CID and EAD fragments from [(Et_3_P)AuGSH]^+^ together with the error in ppm between calculated exact mass and reported experimental *m/z*.

| **Molecular formula** | **Theo m/z** | **Exp m/z** | **ppm** |
| --- | --- | --- | --- |
| [C₁₆H₃₂AuN₃O₆PS]⁺ | 622.1409 | 622.1407 | -0.3 |
| [C₁₆H₃₁AuN₃O₆PS]⁺^•^ | 621.1332 | 621.1297 | -5.6 |
| [C₁₆H₃₀AuN₃O₅PS]⁺ | 604.1298 | 604.1289 | -1.4 |
| [C₁₆H₃₀AuN₃O_6_P]⁺ | 588.1532 | 588.1505 | -4.5 |
| [C₁₅H₃₁AuN₃O₄PS]⁺ ^•^ | 577.1433 | 577.1409 | -4.1 |
| [C₁₄H₂₇AuN₂O₄PS]⁺ | 547.1089 | 547.1078 | -2.0 |
| [C₁₁H₂₅AuN₂O₃PS]⁺ | 493.0984 | 493.0965 | -3.8 |
| [C₁₁H₂₂AuNO₃PS]⁺ | 476.0718 | 476.0706 | -2.5 |
| [C₁₁H₂₁AuNO₃PS]⁺ ^•^ | 475.064 | 475.0632 | -1.7 |
| [C₁₁H₂₅AuN₂O₃P]⁺ | 461.1263 | 461.1258 | -1.1 |
| [C₁₁H₂₂AuNO₃P]⁺ | 444.0997 | 444.099 | -1.5 |
| [C₁₁H₂₃AuN₂O₂P]⁺ | 443.1157 | 443.1139 | -4.0 |
| [C₉H₁₉O₂AuSP]⁺ | 419.0503 | 419.0496 | -1.6 |
| [C₁₀H₂₅AuN₂OP]⁺ | 417.1365 | 417.1353 | -2.8 |
| [C₉H₁₇AuOPS]⁺ | 401.0398 | 401.0391 | -1.7 |
| [C₈H₂₀AuNPS]⁺ | 390.0714 | 390.0701 | -3.3 |
| [C₆H₁₅AuP]⁺ | 315.0577 | 315.0577 | 0.0 |

**Table S2:** free energies values (kJ mol^-1^) of isomers and conformers of [(Et_3_P)AuGSH]^+^ ion relative to **cS-1**, computed in the gas phase at the B3LYP-D3(BJ)/BS1 level of theory.

| **Structure** | **ΔG_rel_** |
| --- | --- |
| cS-1 | 0.0 |
| eN-1 | 0.1 |
| cS-2 | 3.5 |
| cS-3 | 4.5 |
| eN-2 | 5.3 |
| eN-3 | 7.5 |
| eN-4 | 14.5 |
| eCOO-1 | 16.3 |
| eCOO-2 | 20.3 |
| cS-4 | 21.2 |
| eCOO-3 | 23.5 |
| cSH-1 | 35.2 |
| cSH-2 | 38.6 |
| cCO-1 | 42.8 |
| cCO-2 | 44.1 |
| gCOO-1 | 48.7 |
| cSH-3 | 50.2 |
| cCO-3 | 54.5 |
| cSH-4 | 55.2 |
| cSH-5 | 58.3 |
| gCOOH-1 | 59.7 |
| gCOOH-2 | 70.8 |
| gCOOH-3 | 78.6 |
| eCOOH-1 | 85.2 |

**Table S3:** IRMPD absorptions of [(Et_3_P)AuGSH]^+^ ion and calculated vibrational frequencies (cm^-1^) for **cS-1**. Calculated intensities are reported in parentheses (km mol^-1^). Calculated frequencies in the fingerprint (700-1900 cm^-1^) and range are scaled by factors of 0.98. Non relevant modes with intensity lower than 40 km mol^-1^ are omitted.

| **IRMPD**  **frequencies** | **Calculated IR**  **frequencies** | **Assignment** |
| --- | --- | --- |
|  | 704 (52) | ω (αCO)OH Glu, |
| 765 | 772 (65) | δ CH_3_ (Ethyl groups) |
|  | 1063 (46) | ν Cα-NH_3_^+^ Glu, molecular deformation |
|  | 1078 (40) | ν N-Cα Gly, molecular deformation |
| 1143 | 1134 (92) | δs (αCO)OH Gly, δ NH_3_^+^ Glu, δ NH and CH_2_, ν N-C |
|  | 1154 (48) | δs (αCO)OH Glu, δ NH_3_^+^ Glu, δ NH and CH_2_, ν N-C |
|  | 1163 (134) | δs (αCO)OH Glu, δ NH_3_^+^ Glu, δ NH and CH_2_, ν N-C |
|  | 1286 (40) | δs (αCO)OH Glu, δ NH3+ Glu, δ NH and CH_2_, ν N-C |
| 1520 | 1517 (465) | ν_2_ NH_3_^+^ Glu, ν N-C_γ_=O Cys-Glu |
|  | 1533 (217) | ν_2_ NH3+ Glu, ν N-C=O Gly-Cys |
|  | 1540 (81) | ν_2_ NH_3_^+^ Glu, ν N-C=O Gly-Cys |
|  | 1562 (148) | τ NH3+ Glu |
|  | 1627 (160) | ρ NH_3_^+^ Glu |
| 1686 | 1669 (129) | ν C=O Glu |
|  | 1693 (112) | ν C=O Cys and C=O Glu |
| 1785 | 1784 (198) | ν C=O Glu |
|  | 1784 (433) | ν C=O Gly |

ν = stretching mode; δ = bending mode; δ_s_ = scissoring mode; ρ = rocking mode; τ = twisting mode; ω = wagging mode; ν_2_ = umbrella mode.

**Table S4:** IRMPD absorptions of [(Et_3_P)AuGSH]^+^ ion and calculated vibrational frequencies (cm^-1^) for **eN-1**. Calculated intensities are reported in parentheses (km mol^-1^). Calculated frequencies in the fingerprint (700-1900 cm^-1^) and range are scaled by factors of 0.98. Non relevant modes with intensity lower than 40 km mol^-1^ are omitted.

| **IRMPD**  **frequencies** | **Calculated IR**  **frequencies** | **Assignment** |
| --- | --- | --- |
|  | 712 (39) | ρ NH_2_ Glu |
| 766 | 767 (64) | δ CH_2_ and CH_3_ (ethyl tails) |
|  | 1080 (108) | ν NH_2_-Cα Glu |
|  | 1121 (78) | ν Cα-N Cys, ν Cα-N Gly |
|  | 1140 (298) | δ_s_ (CO)OH Gly |
| 1143 | 1149 (49) | δ_s_ (CO)OH Glu, ν Cα-N Cys |
|  | 1159 (207) | δ_s_ (CO)OH Glu, ν Cα-N Cys, ν Cα-N Glu |
|  | 1201 (62) | ω NH_2_, ν CO(Glu)-NH(Cys) |
| 1520 | 1505 (258) | ν CγO(Glu)-NH(Cys), δ N-H Cys |
|  | 1555 (183) | ν CO(Cys)-NH(Gly), δ N-H Gly |
|  | 1603 (128) | δ_s_ NH_2_ |
| 1686 | 1665 (287) | ν C=O Cys |
|  | 1696 (166) | ν Cγ=O Glu |
|  | 1773 (453) | ν C=O Gly |
| 1785 | 1774 (223) | ν C=O Glu |

ν = stretching mode; δ = bending mode; δ_s_ = scissoring mode; ρ = rocking mode; τ = twisting mode; ω = wagging mode.
